# Administration of Anæsthesia

**Published:** 1890-01

**Authors:** 


### Administration of Anæsthetics.

At a meeting of the Brooklyn Surgical Society, June 6, 1889, Dr. Pilcher said: The experience of the past ten years has led me to give preference to that very popular and common inhaler known as the Allis inhaler.

Practically, for the administration of ether, it has seemed to me that the simple cone of Allis, used with that intelligence and care which ought to accompany the administration of an anæsthetic always gives us all we desire, with the one exception of not being economical of the anæsthetic. It is true, I have seen the anæsthetic poured through it onto a patient, which ought not to be, and need not be. Of course a recent graduate, though a fool, will not err in this way in the use of ether with this instrument presented by Prof. Wright; he couldn't pour the ether into the mouth of the patient if he should try. Certainly this is an advantage if to such hands the giving of an anæsthetic must be entrusted. I certainly like this instrument. It seems as simple as any thing of the kind can possibly be and answer to the indications.

I am convinced that in a very considerable portion of cases the use of ether as an anæsthetic is dangerous, and have been rejoiced to see the tendency, which is so marked in the profession at present, to study the advantages and dangers of different anæsthetics with regard to different patients, and to suit to particular patients the anæsthetics which experience has shown to be the best adapted for them; so that there is rapidly becoming less partisanship with regard to this or that anæsthetic. It is a marked feature for which I think all thoughtful surgeons should rejoice in the conditions of to-day. The long administration of ether is certainly depressing. Even the short administration of ether has a more or less irritating effect upon the respiratory passages. Ether to any considerable extent has also an irritating effect upon the urine-secreting apparatus, and it is certainly wise for us to take these things into account in choosing our anæsthetic, and if for any reason we feel we must use ether as an anæsthetic, it seems especially wise that we should take advantage of some such device as this which has been presented to us this evening, which would diminish as much as possible the irritating effect of that particular anæsthetic.

In many cases I am convinced that chloroform should be used as the anæsthetic rather than ether; in all cases where there is recognizable irritation of the bronchial and pulmonary tissues, also where there is a diseased condition of the urinary secretive apparatus; that in young children, likewise, who as a class have been shown by experience to be peculiarly pleasantly affected by chloroform, it should be used; and at the other extreme of life, in the aged, who as a class either already suffer from, or are on the verge of, renal and pulmonary degenerative changes.

Now, as to the methods of administering chloroform: The most convenient and safe inhaler is always at command in a common tumbler or a common teacup, into the bottom of which a handkerchief can be put so that it shall be an inch or two away from the nostrils or mouth, and which, when put over the mouth and nose can not be forced so close down upon the cheek, upon either side, that there will not always remain still considerable apertures at those points for the abundant admission of air with the chloroform vapor. This is nothing new, but I have had occasion so many times to suggest it to men of large experience and of age in the profession, whom I have seen attempting to administer chloroform in both wasteful and dangerous methods, that possibly it may not be out of place to notice this method in this connection.—*Brooklyn Med. Jour.*, Nov. 1889.

